# c-Jun Amino-Terminal Kinase-1 Mediates Glucose-Responsive Upregulation of the RNA Editing Enzyme ADAR2 in Pancreatic Beta-Cells

**DOI:** 10.1371/journal.pone.0048611

**Published:** 2012-11-06

**Authors:** Liu Yang, Ping Huang, Feng Li, Liyun Zhao, Yongliang Zhang, Shoufeng Li, Zhenji Gan, Anning Lin, Wenjun Li, Yong Liu

**Affiliations:** 1 Key Laboratory of Nutrition and Metabolism, Institute for Nutritional Sciences, Shanghai Institutes for Biological Sciences, Chinese Academy of Sciences, Shanghai, China; 2 State Key Laboratory of Cell Biology, Institute of Biochemistry and Cell Biology, Shanghai Institutes for Biological Sciences, Chinese Academy of Sciences, Shanghai, China; Pennington Biomedical Research Center, United States of America

## Abstract

A-to-I RNA editing catalyzed by the two main members of the adenosine deaminase acting on RNA (ADAR) family, ADAR1 and ADAR2, represents a RNA-based recoding mechanism implicated in a variety of cellular processes. Previously we have demonstrated that the expression of ADAR2 in pancreatic islet β-cells is responsive to the metabolic cues and ADAR2 deficiency affects regulated cellular exocytosis. To investigate the molecular mechanism by which ADAR2 is metabolically regulated, we found that in cultured β-cells and primary islets, the stress-activated protein kinase JNK1 mediates the upregulation of ADAR2 in response to changes of the nutritional state. In parallel with glucose induction of ADAR2 expression, JNK phosphorylation was concurrently increased in insulin-secreting INS-1 β-cells. Pharmacological inhibition of JNKs or siRNA knockdown of the expression of JNK1 prominently suppressed glucose-augmented ADAR2 expression, resulting in decreased efficiency of ADAR2 auto-editing. Consistently, the mRNA expression of *Adar2* was selectively reduced in the islets from JNK1 null mice in comparison with that of wild-type littermates or JNK2 null mice, and ablation of JNK1 diminished high-fat diet-induced *Adar2* expression in the islets from JNK1 null mice. Furthermore, promoter analysis of the mouse *Adar2* gene identified a glucose-responsive region and revealed the transcription factor c-Jun as a driver of *Adar2* transcription. Taken together, these results demonstrate that JNK1 serves as a crucial component in mediating glucose-responsive upregulation of ADAR2 expression in pancreatic β-cells. Thus, the JNK1 pathway may be functionally linked to the nutrient-sensing actions of ADAR2-mediated RNA editing in professional secretory cells.

## Introduction

RNA editing through the hydrolytic C6 deamination of adenosine (A) to yield inosine (I) represents a pivotal post-transcriptional mechanism that further diversifies the cellular transcriptome and proteome [1], [2], [3]. Based upon the RNA substrates that have been found to undergo A to I editing within regions with double-stranded (ds) structural character, this genetic recoding process has been implicated in the functional modifications of proteins [1], [2], [3], [4], [5], alternative splicing [6], and microRNA biogenesis [7]. A growing body of evidence has established that A to I RNA editing plays essential roles in the function and development of the central nervous system, largely through editing of transcripts encoding the neurotransmitter receptors and ion channels, including the ionotropic glutamate receptors (GluRs), G-protein-coupled serotonin-2C subtype receptor, and Kv1.1 potassium channel [4], [8], [9], [10], [11].

In mammals, two members of the adenosine deaminase acting on RNA (ADAR) family, ADAR1 and ADAR2, are enzymatically active for catalyzing the A to I deamination reaction [12]. Both ADAR1 and ADAR2 are ubiquitously expressed in many tissues [13], [14], [15]. Multiple promoters have been identified to control the expression of ADAR1, generating transcripts with alternative exon 1 structures that encode two ADAR1 forms, an interferon (IFN)-inducible protein of ∼150 kDa and a constitutively expressed N-terminally truncated protein of ∼110 kDa [16], [17], [18]. In addition to the regulatory elements found within the IFN-inducible ADAR1 promoter [19], [20], recent studies revealed distinct tissue-specific expression features for different ADAR1 transcripts [21]. In contrast, the promoter that directs the ADAR2 expression has not been functionally characterized, despite that a putative promoter region upstream of a newly identified exon was described for both human and mouse ADAR2 genes [22]. While it is yet to be established whether ADAR2 possesses multiple promoters like ADAR1 to produce multiple transcripts, it also remains unclear if regulatory mechanism(s) exists for the transcriptional control of ADAR2 in a tissue- or cell type-specific fashion.

Many intracellular signaling mechanisms act to modulate the function of pancreatic β-cells, which play a central role in glucose homeostasis through fuel-regulated secretion of insulin [23]. Glucose, the primary physiological stimulator of insulin synthesis and secretion, has been shown to trigger the activation of c-Jun amino-terminal kinase (JNK) [24], the stress-activated protein kinase that belongs to the large mitogen-activated protein kinase (MAPK) family [25]. The JNK pathway is known to integrate signals from a diversity of extracellular stimuli and regulate various cellular processes such as survival, proliferation and apoptosis [25]. Among the three JNK isoforms, JNK1 and JNK2 are found to be ubiquitously expressed, while JNK3 is mainly expressed in brain, pancreatic islets, testis and heart [26]. For JNK1 and JNK2, alternative splicing yields multiple protein forms of ∼54 kDa and ∼46 kDa [27]. Distinct intracellular mechanisms are operational in mounting cell- or stimulus-specific responses that result in JNK activation, which phosphorylates and activates transcription factors including the c-Jun component of the activating protein-1 (AP-1) [28]. Documented studies have implicated the JNK pathway in metabolic dysregulation associated with obesity, insulin resistance, and type 2 diabetes [29], [30]. In pancreatic β-cells, JNK is thought to be involved in suppression of insulin gene expression under oxidative stress [31] and cytokine-induced apoptosis [32]. While the JNK isoforms may possess functional redundancy in mediating cellular adaptation responses to various stress stimuli, it remains poorly understood whether JNK1 or JNK2 is specifically linked to regulation of different cellular aspects of the nutrient-sensing actions in β-cells.

Previously we demonstrated that RNA editing by ADAR2 is upregulated selectively in the pancreatic islets of obese mice when chronically fed a high-fat diet, and ADAR2 expression can be stimulated by glucose in the rat insulinoma INS-1 β-cells [33]; moreover, knockdown of the expression of ADAR2 in pancreatic islets and INS-1 cells leads to attenuation of glucose-stimulated insulin secretion, suggesting that ADAR2 is coupled to cellular exocytosis in professional secretory cells [34]. However, the molecular mechanism for the nutritional regulation of ADAR2 expression in β-cells is largely unclear. Here we examined the mechanistic link between glucose-stimulated JNK activation and ADAR2 expression. Our findings indicate that JNK1 serves as a crucial component in mediating glucose-responsive upregulation of ADAR2 in pancreatic β-cells.

## Materials and Methods

### Cell Lines and Pancreatic Islet Isolation

The rat insulinoma INS-1 cell line [35], a generous gift from Dr. Christopher B. Newgard (Duke University Medical Center), were maintained in RPMI 1640 containing 11.1 mM D-glucose, 10% fetal calf serum, penicillin (100 U/ml) and streptomycin (100 mg/ml), 10 mM Hepes, 2 mM L-glutamine, 1 mM sodium pyruvate, and 0.05 mM 2-β-mercaptoethanol. Cells with passage numbers of 19 to 30 were used. Pancreatic islets were isolated from mice at 14–24 weeks of age using the Liberase (Roche) digestion method as previously described in detail [33].

### Antibodies, Western Immunoblot, Expression Plasmids and Chemical Reagents

Goat polyclonal antibodies against ADAR2 (C-15) and ADAR1 (L-15) were purchased from Santa Cruz Biotechnology. JNK [pThr^183^/Tyr^185^], JNK, c-Jun [pSer^63^], c-Jun, AKT [pSer^473^] and AKT antibodies were from Cell Signaling. Monoclonal antibodies against β-actin and α-tubulin were purchased from Sigma and Biomeda (Scottsdale, AZ, USA), respectively. For Western immunoblotting, cellular protein extracts were prepared and separated by SDS-polyacrylamide gel electrophoresis (PAGE) and transferred to polyvinylidene difluoride membrane filter (Amersham Biosciences). After incubation with the desired antibodies, the blots were developed with Thermo Scientific’s SuperSignal West Pico Chemiluminescent Substrate. The expression vectors for c-Jun and JunB were generously provided by Dr. Jing Fang (the Institute for Nutritional Sciences, SIBS, CAS). SP600125, 2-deoxyglucose, EGTA, 2-APB and somatostatin-14 were purchased from Sigma; hydroxy-2-naphthalenylmethylphosphonic acid tris acetoxymethyl ester [HNMPA(AM)_3_] was from Alexis Biochemicals.

### RT-PCR Analysis

Total RNA was isolated from INS-1 cells with TRIzol (Invitrogen) or from primary islets with RNeasy Plus Mini Kit (Qiagen). After treatment with RNase-free DNase I (Roche Applied Science), first-strand cDNA was synthesized with M-MLV Reverse Transcriptase (Invitrogen). Quantitative real-time PCR was conducted using an ABI 7500 Fast Real-Time PCR System and Power SYBR Green PCR Master Mix (Applied Biosystems) following the manufacturer’s recommendations. *Cyclohphilin* or *Gapdh* was used as an internal control for normalization. Primers used for each target gene were listed in Table S1. For quantitative analysis of ADAR2 auto-editing, regular PCR was performed using the TaKaRa Taq Kit (Takara). After analysis by 3.0% agarose gel electrophoresis, editing efficiency was quantified using the Quantity One software (Bio-Rad).

### Generation of Recombinant Adenoviruses and Viral Infection

Recombinant adenoviruses Ad-shJNK1 and Ad-shLacZ, which express shRNAs directed against rat JNK1 and LacZ genes, respectively, were generated using the BLOCK-iT ™ Adenoviral RNAi Expression System (Invitrogen, Carlsbad, CA) as previously described [34]. The adenovirus used for JNK1 knockdown contains the following target sequence: 5′ -GCAGAAGTAAACGTGACAACA-3′. The viral titers were determined by the tissue culture infectious dose 50 (TCID50) method. INS-1 cells were infected by adenoviruses at an MOI of 20 for 72 hours.

### Animal Studies

All experimental protocols were approved by the Institutional Animal Care and Use Committee at the Institute for Nutritional Sciences, Shanghai Institutes for Biological Sciences, Chinese Academy of sciences. *Jnk1^−/−^* and *Jnk2^−/−^* mice were housed in laboratory cages at a temperature of 23±3°C and a humidity of 35±5% under a 12 h dark/light cycle (lights on at 6∶30 a.m.) in accredited animal facilities at the Shanghai Institutes for Biological Sciences, CAS. Heterozygote mice were subsequently intercrossed to yield homozygotes and wild-type littermates. For diet-induced obesity, female mice maintained on a normal chow diet were fed *ad libitum* a low-fat diet (LFD, 10 kcal% fat) or high-fat diet (HFD, 60 kcal% fat) (Research Diets, Inc., New Brunswick, NJ). The body weight was monitored monthly. Total body fat content was measured by nuclear magnetic resonance (NMR) using the Minispec Mq7.5 (Bruker, Germany). Fasting glucose was determined in blood collected from tail vein of female mice at 24 weeks after a 6 hours fasting (from 8∶30–14∶30) using a glucometer (FreeStyle, Alameda, California, USA).

### Mouse *Adar2* Promoter Constructs and Luciferase Reporter Assays

The promoter of mouse *Adar2* gene, which corresponds to the region of -4921 to +186 with respect to the putative transcription start site (denoted nucleotide +1), was amplified by PCR using mouse genomic DNA as the template. The DNA fragment was subsequently subcloned into plasmid pGL3-basic (Promega, Madison, WI) via *Mlu* I and *Xho* I restriction sites to generate the P*_Adar2_*-4921-Luc construct. Three constructs with 5′-terminal deletions of the promoter, P*_Adar2_*-1256-Luc, P*_Adar2_*-219-Luc, and P*_Adar2_*-63-Luc were derived from P*_Adar2_*-4921-Luc. A PCR-based mutagenesis strategy was used to generate the mutant P*_Adar2_*-219-Luc with a four-nucleotide substitution within the AP1 site, using the Muta-direct site-directed mutagenesis kit (SBS Genetech) and the following primers: 5′-CAGGAGGCGCGCATTGAAGCGTGGAGCGCT-3′ (forward) and 5′ -AGCGCTCCACGCTTCAATGCGCGCCTCCTG-3′ (reverse). Rat *Insulin I* promoter reporter construct (410RIP1-Luc) was kindly provided by Dr. M. German (University of California at San Francisco, Medical School). INS-1 cells with 40–60% confluence were maintained at 2.8 mM glucose for 6 hours before transfection using Lipofectamine 2000 (Invitrogen, Grand Island, NY). Renilla luciferase reporter plasmid pRL-TK (Promega, Madison, WI) was used as an internal control. Cells were treated at 24 hours after transfection with 16.7 mM glucose for 16–24 hours, and the luciferase activities were measured using Dual-luciferase Assay Kit (Promega, Madison, WI) according to the manufacturer’s instructions.

### Statistical Analysis

Statistical analysis was performed with unpaired two-tailed t-test or one-way or two-way analysis of variance (ANOVA). p<0.05 was considered to be statistically significant.

## Results

### Glucose-stimulated JNK Phosphorylation Accompanies Upregulation of ADAR2 in INS-1 β-cells

**Figure 1:**
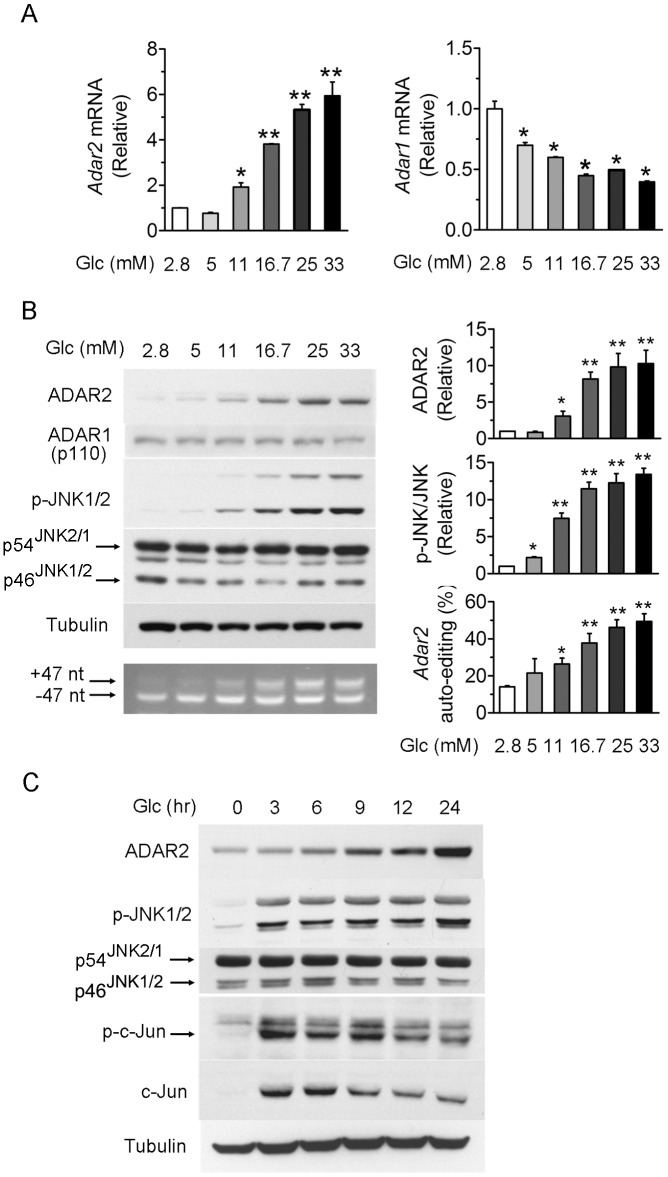
Glucose-induced ADAR2 expression is accompanied by concomitant phosphorylation of JNK. (**A–B**) INS-1 β-cells were maintained at 2.8 mM glucose (Glc) for 18 hours and were subsequently cultured for 24 hours in serum-free medium with glucose at the indicated concentrations. (**A**) The mRNA abundance of *Adar2* and *Adar1* (both the p150 and p110 isoforms) was determined by real-time quantitative RT-PCR. Data represent the mean±SEM from three independent experiments. *P<0.05, **P<0.01 versus control cells cultured at 2.8 mM glucose. (**B**) Cell lysates were analyzed by immunoblotting using the indicated antibodies. Tubulin was used as a loading control. Phosphorylated JNK (p-JNK) was detected with anti-JNK (pThr183/pTyr185) antibody. The efficiency of ADAR2 auto-editing was measured by RT-PCR assessment of the editing-dependent alternative splicing event. The PCR products corresponding to transcripts with or without the 47-nucleotide insert are indicated. Data are representative of three independent experiments. Relative ADAR2 protein expression levels, p-JNK/JNK ratios, and ADAR2 auto-editing efficiencies were determined from densitometric quantifications, all shown as the mean±SEM (n = 3 independent experiments). *P<0.05, **P<0.01 versus control cells cultured at 2.8 mM glucose. (**C**) INS-1β-cells maintained at 2.8 mM glucose for 12 hours were pre-cultured without serum at 2.8 mM glucose for 6 hours and then stimulated with 16.7 mM glucose for the indicated time intervals. Cell lysates were analyzed by immunoblotting using the indicated antibodies. Shown are representative immunoblots from three independent experiments. Phosphorylated c-Jun (p-c-Jun) was detected by anti-c-Jun (pSer63) antibody.

To investigate the signaling mechanism by which ADAR2 is regulated in β-cells, we first tested the association of JNK activation with glucose-dependent upregulation of ADAR2. Consistent with our previous finding that *Adar2* is a glucose-inducible gene [33], glucose stimulation dose-responsively increased the mRNA abundance of *Adar2* but not *Adar1* in INS-1 β-cells, as shown by quantitative RT-PCR analysis (Fig. 1A). Western immunoblotting also revealed significant elevations of ADAR2 protein but not the p110 form of ADAR1 protein in response to increasing glucose concentrations (Fig. 1B). This corresponded with increased efficiency of auto-editing of *Adar2* transcripts (Fig. 1B), as determined by RT-PCR that detected the 47-nucleotide insertion resulting from ADAR2 editing-initiated alternative splicing [6]. Interestingly, glucose stimulation of ADAR2 expression and RNA editing was concomitantly accompanied by elevated phosphorylation of JNK1/2 at Thr^183^/Tyr^185^ (Fig. 1B). Furthermore, the time-course analysis showed that stimulation by glucose of ADAR2 protein expression for different time intervals was also paralleled with induced JNK1/2 phosphorylation as well as increased phosphorylation of c-Jun (Fig. 1C), the major target of JNK kinases. Notably, the protein level of c-Jun was also augmented upon glucose stimulation (Fig. 1C), which could be largely ascribable to JNK phosphorylation-dependent enhancement of c-Jun protein stability [36]. These results raised the possibility that the JNK pathway is coupled to glucose regulation of ADAR2-mediated RNA editing in β-cells.

### JNK Activation-associated ADAR2 Upregulation in β-cells is a Glucose-sensing Event

**Figure 2:**
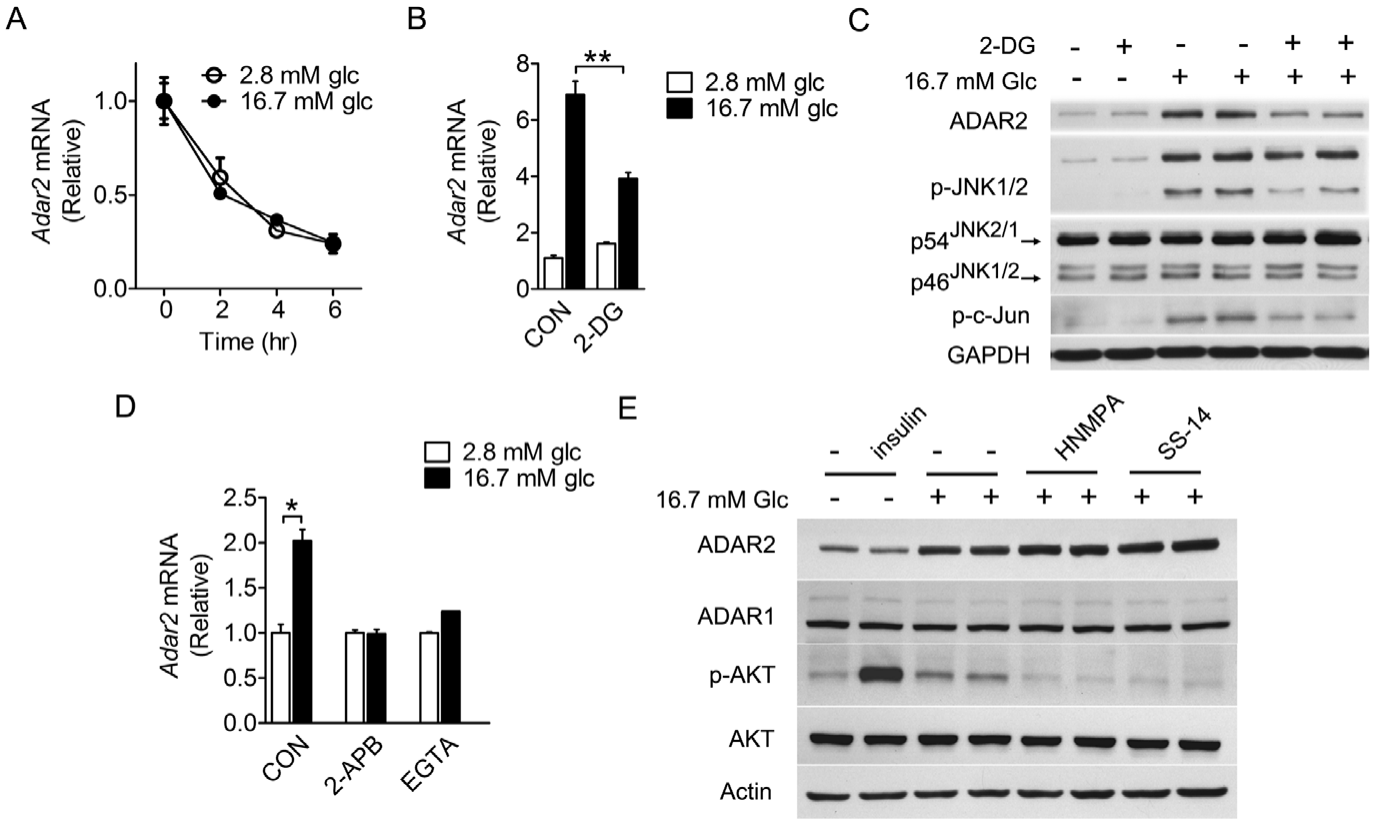
Blocking glucose metabolism and calcium mobilization blunts glucose stimulation of JNK phosphorylation and ADAR2 expression. (**A**) High glucose did not affect the stability of *Adar2* mRNA transcripts. INS-1 cells cultured at 16.7 mM glucose for 3 hours were maintained at 2.8 or 16.7 mM glucose in the presence of 5 µg/ml actinomycin D for the indicated time intervals. The abundance of *Adar2* mRNA was analyzed by real-time RT-PCR. Results represent the mean±SEM from three independent experiments. (**B–C**) INS-1 cells maintained at 2.8 mM glucose for 12 hours were pre-cultured without serum at 2.8 mM glucose for 6 hours and then stimulated with 16.7 mM glucose in the absence or presence of 20 mM 2-deoxyglucose (2-DG, an inhibitor of glucose glycolysis) for 24 hours. (**B**) The abundance of *Adar2* mRNA was determined by real-time RT-PCR. Data are shown as the mean±SEM from three independent experiments. **P<0.01 versus control cells at 2.8 mM glucose. (**C**) Immunoblotting was performed using the indicated antibodies. Results are representative of three independent experiments. (**D**) INS-1 cells pre-cultured at 2.8 mM glucose for 18 hours were incubated with 5 mM EGTA (a blocker of calcium influx) or 50 µM 2-APB (an inhibitor of ER calcium release through the IP3 receptor) for 30 min before stimulation by 16.7 mM glucose for 3 hours. The abundance of *Adar2* mRNA was determined by real-time RT-PCR. Data are shown as the mean±SEM from three independent experiments. *P<0.05 versus control cells at 2.8 mM glucose. (**E**) INS-1 cells maintained at 2.8 mM glucose for 12 hours were pre-cultured without serum at 2.8 mM glucose for 6 hours and then treated for 24 hours with 100 nM insulin, or with 16.7 mM glucose in the absence or presence of 10 µM HNMPA-(AM) 3 (an inhibitor of insulin receptor tyrosine kinase activity) or 500 nM somatostatin-14 (SS-14, an inhibitor of insulin secretion). The inhibitors were added 30 min before glucose stimulation. Cell lysates were analyzed by immunoblotting with the indicated antibodies. Shown are representative results of three independent experiments.

As glucose-induced upregulation of ADAR2 and the concurrent JNK activation in INS-1 cells may represent a glucose-sensing event, we asked if glucose can affect the stability of *Adar2* mRNA or if glucose metabolism is required for the stimulation of ADAR2 expression and JNK phosphorylation. Upon treatment with actinomycin D for up to 6 hours, comparable decreases in the *Adar2* mRNA abundance were detected by quantitative RT-PCR in INS-1 cells when maintained at 2.8 or 16.7 mM glucose (Fig. 2A), suggesting that glucose had no effect upon the *Adar2* mRNA decay and glucose augmentation of the *Adar2* mRNA level may reflect an induction of its transcription. Moreover, inhibition of glucose glycolysis in INS-1 cells with 2-deoxyglucose (2-DG), the non-metabolizable glucose analogue [37], not only blunted glucose induction of the ADAR2 mRNA and protein expression (Fig. 2B–C), but also attenuated glucose stimulation of JNK and c-Jun phosphorylation (Fig. 2C). Next, we examined whether mobilization of intracellular calcium, a critical cellular signaling event that mediates glucose-regulated insulin secretion in β cells [38], is also involved. Indeed, blocking calcium influx with EGTA or calcium release from the endoplasmic reticulum with 2-aminoethoxydiphenyl borate (2-APB) significantly diminished glucose-dependent upregulation of *Adar2* mRNA expression (Fig. 2D). However, it is unclear how the calcium-triggered downstream signaling events are linked to regulation of ADAR2. To test whether an indirect autocrine action from glucose-stimulated insulin secretion is involved, we examined the effects of insulin or inhibitors of insulin signaling or secretion in INS-1 cells. Whereas insulin treatment of cells cultured at 2.8 mM glucose did not show a stimulatory effect upon the expression of ADAR2 protein, inhibition of insulin signaling with HNMPA-(AM)_3_ or its secretion with somatostatin-14 in cells cultured at 16.7 mM glucose had no detectable effects on glucose-stimulated expression of ADAR2 protein (Fig. 2E). Together, these data indicate that the upregulation of ADAR2 is selective to glucose stimulation and directly relies on glucose metabolism in β-cells, and JNK activation may be an integral part of this glucose-sensing event.

### Inhibition of JNK Activity Abrogates Glucose Enhancement of ADAR2 Expression and Auto-editing

**Figure 3:**
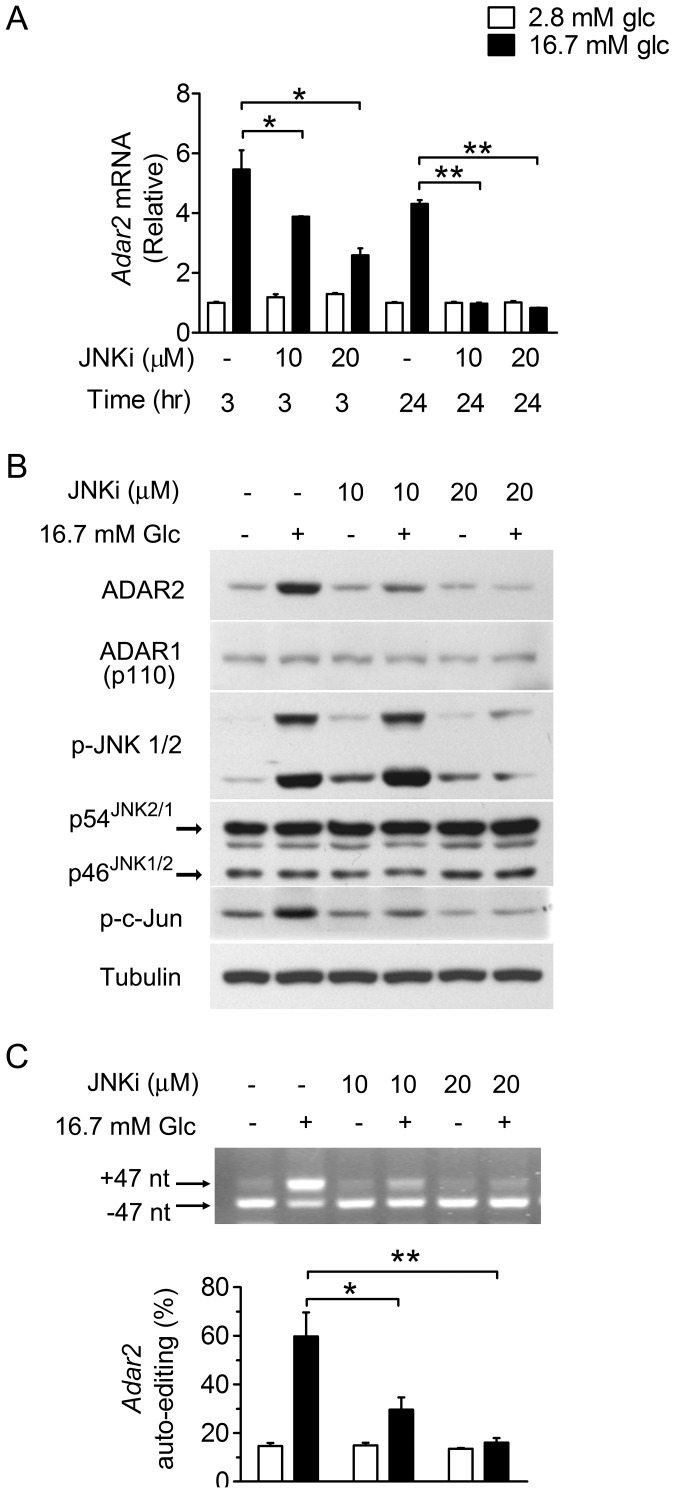
Inhibition of JNK activity reduces glucose-stimulated ADAR2 expression and auto-editing in β-cells. INS-1 cells maintained at 2.8 mM glucose for 12 hours were cultured without serum for 6 hours at 2.8 mM glucose before pretreatment for 30 min with DMSO (−) or 10 or 20 µM JNK inhibitor SP600125 (JNKi). Cells were then cultured for 3 or 24 hours at 2.8 or 16.7 mM glucose in the presence of DMSO or JNKi at 10 or 20 µM as indicated. (**A**) The relative mRNA abundance of *Adar2* was determined by quantitative RT-PCR. Data represent the mean±SEM from three independent experiments. *P<0.05, **P<0.01. (**B**) Lysates from cells treated for 24 hours were analyzed by immunoblotting using the indicated antibodies. Results are representative of three independent experiments. (**C**) The efficiency of ADAR2 auto-editing was analyzed by RT-PCR for cells treated for 24 hours. Densitometric quantifications are shown as the mean±SEM (n = 3 independent experiments). *P<0.05, **P<0.01.

To determine if the JNK pathway is critical for glucose-stimulated ADAR2 expression and ADAR2-mediated RNA editing, we evaluated the impact of SP600125, a specific inhibitor of JNK activity (JNKi) [39]. Glucose-stimulated expression of *Adar2* mRNA was reduced by ∼30% and ∼55%, respectively, in INS-1 cells that were cultured at 16.7 mM glucose for 3 hours in the presence of 10 µM and 20 µM JNKi, while it was totally blocked in cells when cultured for 24 hours (Fig. 3A). Treatment of INS-1 cells with 10 µM or 20 µM JNKi for 24 hours did not affect the expression of ADAR1-p110 protein but resulted in partial (by ∼65%) or complete abrogation of glucose-induced increases in the level of ADAR2 protein; these were accompanied by partial (∼50%) or total inhibition of glucose-stimulated c-Jun phosphorylation (Fig. 3B). Consistent with the reported findings [39], we also observed a marked decrease in JNK phosphorylation when cells were treated with 20 µM JNKi (Fig. 3B). In parallel, analysis of ADAR2 auto-editing showed that treatment with JNKi for 24 hours markedly reduced the stimulation by glucose of ADAR2-catalyzed RNA editing (Fig. 3C). These results thus suggest that the JNK pathway may play a crucial role in mediating the glucose-responsive upregulation of ADAR2 expression and ADAR2-associated RNA editing.

### Selective Knockdown of JNK1 Attenuates Glucose-stimulated ADAR2 Expression

**Figure 4:**
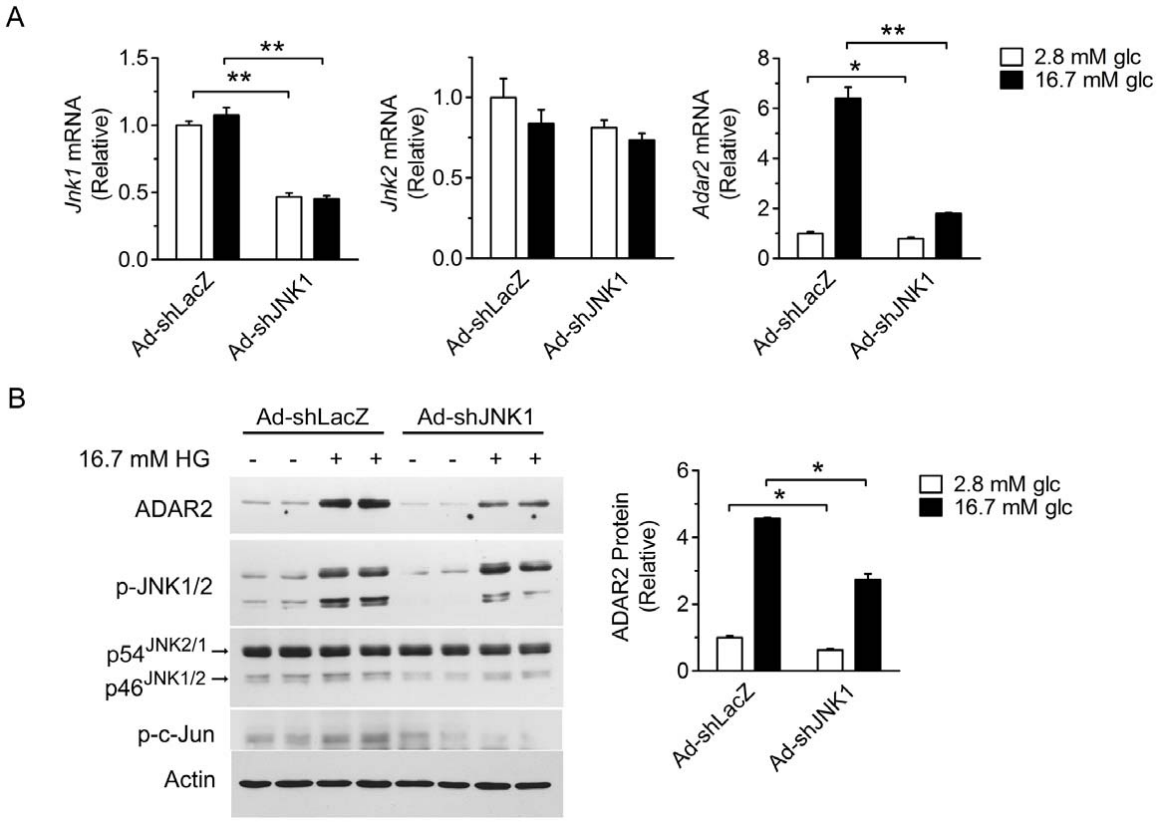
Knockdown of the expression of JNK1 attenuates glucose-induced ADAR2 expression in β-cells. INS-1 cells were infected with recombinant adenoviruses Ad-shLacZ or Ad-shJNK1 at an MOI of 20 for 72 hours and maintained at 2.8 mM glucose for 18 hours. Cells were subsequently cultured in serum-free medium with 2.8 mM or 16.7 mM glucose for 24 hours. (**A**) The mRNA expression levels of *Jnk1, Jnk2* and *Adar2* were determined by quantitative RT-PCR. Data represent the mean±SEM from three independent experiments. *P<0.05, **P<0.01 versus cells infected with the Ad-shLacZ control virus. (**B**) Immunoblot analysis of cell lysates using the indicated antibodies. Actin was used as a loading control. The relative level of ADAR2 protein was determined from densitometric quantifications, shown as the mean±SEM (n = 3 independent experiments).

To rule out the possible nonspecific effect of the JNK inhibitor and investigate which JNK isoform is specifically involved, we employed the recombinant adenovirus Ad-shJNK1 expressing a small hairpin (sh) RNA directing against the coding region of *Jnk1* to knock down its expression. In comparison with the control virus Ad-shLacZ, Ad-shJNK1 infection of INS-1 cells significantly decreased the mRNA expression level of *Jnk1*, but did not affect that of *Jnk2* (Fig. 4A). Furthermore, Ad-shJNK1-mediated knockdown of JNK1 markedly reduced (by ∼70%) the induction of *Adar2* mRNA expression in response to stimulation by glucose at 16.7 mM (Fig. 4A; from 6.4±0.44-fold to 1.8±0.03-fold). Consistently, Western immunoblot analysis showed that knockdown of JNK1 expression in INS-1 cells infected with Ad-shJNK1 blunted the phosphorylation of c-Jun and significantly decreased (by ∼45%) the protein expression level of ADAR2 upon stimulation by 16.7 mM glucose (Fig. 4B). These data indicate that JNK1 may be an important player that contributes to the glucose-dependent upregulation of ADAR2 in β-cells.

### JNK1 is a Critical Driver of ADAR2 Expression in Pancreatic Islets

**Figure 5:**
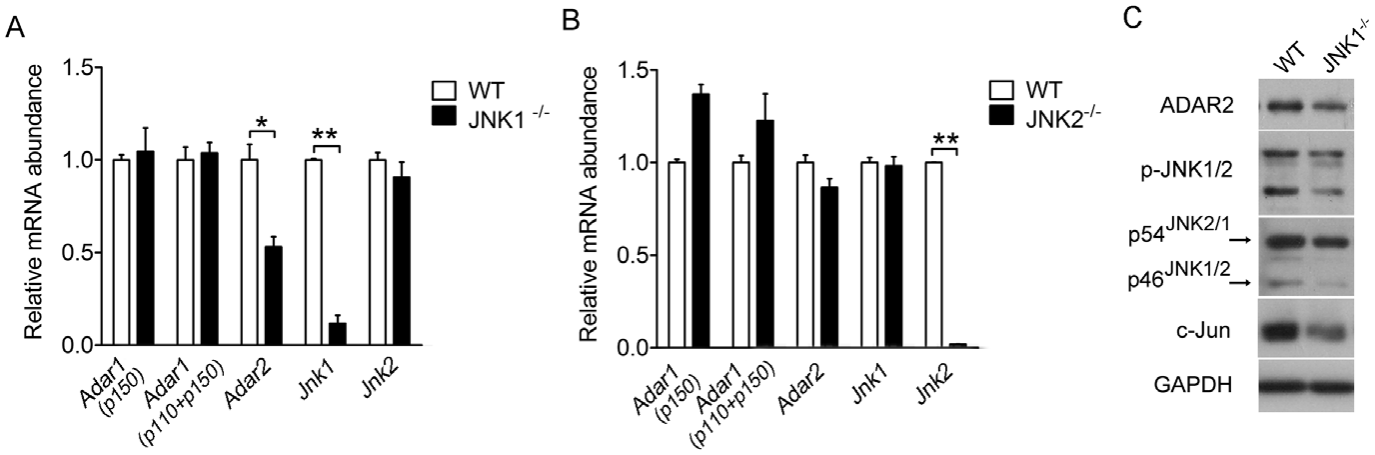
ADAR2 expression is decreased in the islets of JNK1 knockout mice. (**A–B**) Primary islets were isolated from male (**A**) JNK1^−/−^ (n = 5) or (**B**) JNK2^−/−^ (n = 4) knockout mice, or from their wild type (WT) littermates (n = 5). The mRNA abundance of *Adar1 (p150)*, *Adar1 (p150 and p110), Adar2, Jnk1* and *Jnk2* was analyzed from pooled islets by real-time RT-PCR. Data represent the mean±SEM. *P<0.05, **P<0.01 versus WT islets. (**C**) Primary islets from male JNK1^−/−^ or WT mice were analyzed by immunoblotting using the indicated antibodies. GAPDH was used as a loading control. Shown are representative data from three mice for each genotype.

To further determine if JNK1 is specifically essential for regulating the expression of ADAR2 in β-cells, we measured the mRNA expression of *Adar2* in the primary islets isolated from mice with whole-body deletion of the *Jnk1* or *Jnk2* gene [29]. As shown by quantitative RT-PCR assessment, the mRNA expression of *Adar2* displayed a significant reduction (by ∼50%) in the islets of JNK1^−/−^ mice (Fig. 5A) but not JNK2^−/−^ mice (Fig. 5B). By contrast, the expression levels of *Adar1* mRNAs, including those encoding the IFN-inducible p150 form or both the p150 and p110 forms, did not exhibit significant alterations (Fig. 5). Moreover, Western immunoblot assessment showed that the protein expression level of ADAR2 was apparently decreased in the islets of JNK1^−/−^ mice (Fig. 5C), which was accompanied by reduced protein level of c-Jun expression. Therefore, JNK1 is likely to serve as a critical driver of ADAR2 expression in pancreatic islets.

### High-fat Diet-induced Expression of ADAR2 is Dependent on JNK1 in Pancreatic Islets

**Figure 6:**
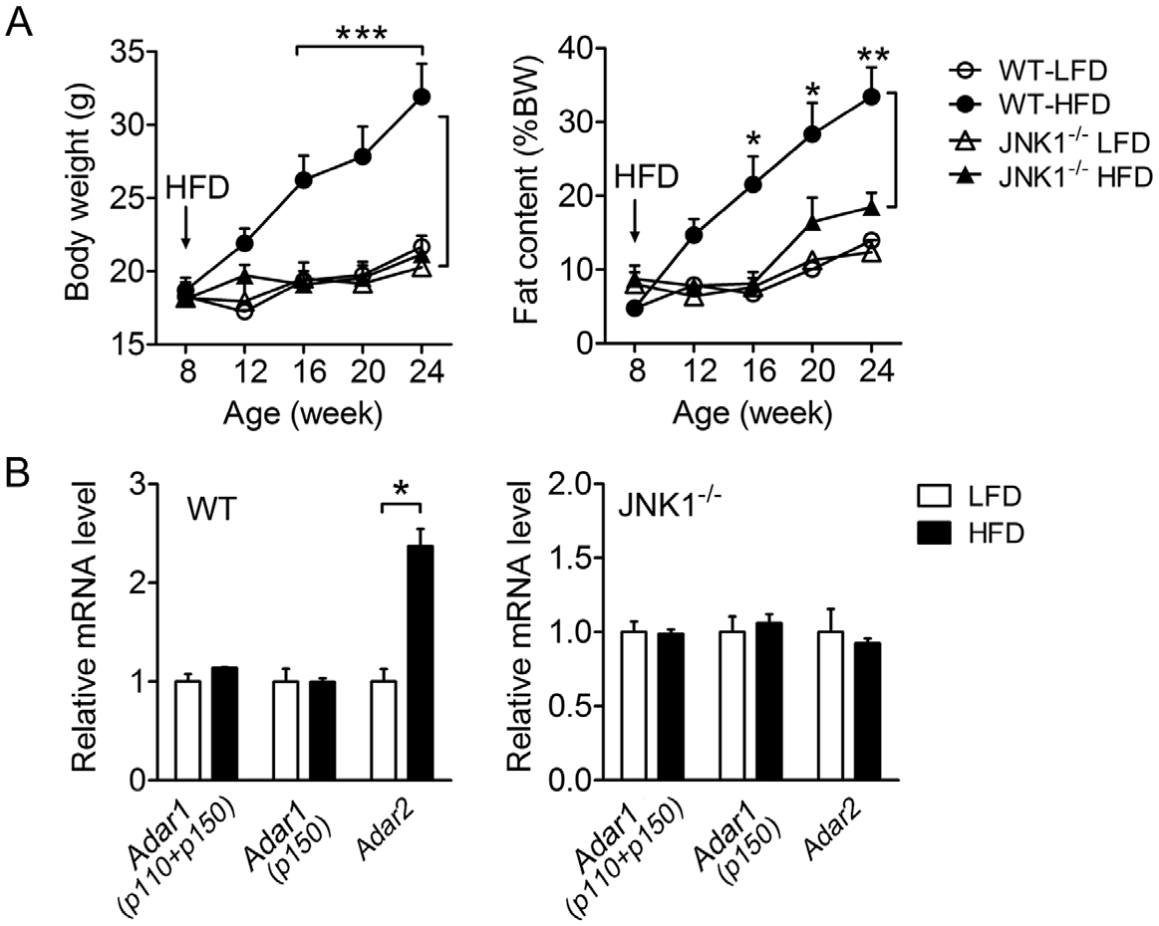
Ablation of JNK1 abrogates obesity-associated upregulation of ADAR2 in the islets of mice fed a high-fat diet. Female WT or JNK1^−/−^ mice (n = 5-7/group) at 8 weeks of age were fed a low-fat diet (LFD) or a high-fat diet (HFD) for 16 weeks. (**A**) The body weight and fat content were monitored. Data are shown as the mean± SEM. *P<0.05, **P<0.01, ***P<0.001 versus LFD-fed WT littermates. (**B**) The mRNA abundance of *Adar1 (p150)*, *Adar1 (p150 and p110)*, and *Adar2* was assessed from the isolated primary islets by quantitative RT-PCR (n = 4/group). *Cyclophilin* was used as an internal control, and the relative expression level for each gene was normalized to that from LFD-fed mice for each genotype (set as 1.0). Data are shown as the mean±SEM. *P<0.05 versus LFD-fed WT mice.

We have previously reported that *Adar2* transcripts were selectively upregulated in the pancreatic islets isolated from the insulin-resistant obese mice which were chronically fed a high-fat diet (HFD) [33]. We then asked if deficiency in JNK1 signaling could affect the upregulation of islet *Adar2* in response to HFD feeding. When challenged with HFD for up to 24 weeks, the wild-type (WT) littermates exhibited substantial increases in their body weight gain and whole-body fat content as compared with the low-fat diet (LFD)-fed control mice (Fig. 6A), which was associated with elevated blood glucose levels in the fasted state (Fig. S1). In contrast, JNK1^−/−^ mice displayed marked resistance to the HFD-induced obesity (Fig. 6A) as well as normalized fasting glucose levels (Figure S1). This is in accordance with the documented studies demonstrating the crucial role of JNK1 in protecting mice against diet-induced obesity and insulin resistance [29]. Importantly, quantitative RT-PCR analysis showed that the expression of *Adar2* mRNA was augmented by ∼2-fold in the islets from HFD-fed WT mice, which was abolished in JNK1-deficient islets (Fig. 6B). On the other hand, JNK1 ablation led to no significant changes of islet *Adar1* mRNA expression, which did not respond to HFD feeding (Fig. 6B). Hence, JNK1 is a key component that mediates the metabolic regulation of *Adar2* in the islets in response to overnutrition. Moreover, as ADAR2 knockdown could lead to impaired insulin exocytosis [34], the failure of ADAR2 upregulation in the islets of HFD-fed JNK1^−/−^ mice may contribute to the lowered circulating insulin level that was reported for the JNK1^−/−^ mice when fed HFD [29].

### Characterization of the Glucose-inducible Promoter of Mouse *Adar2* Gene

**Figure 7:**
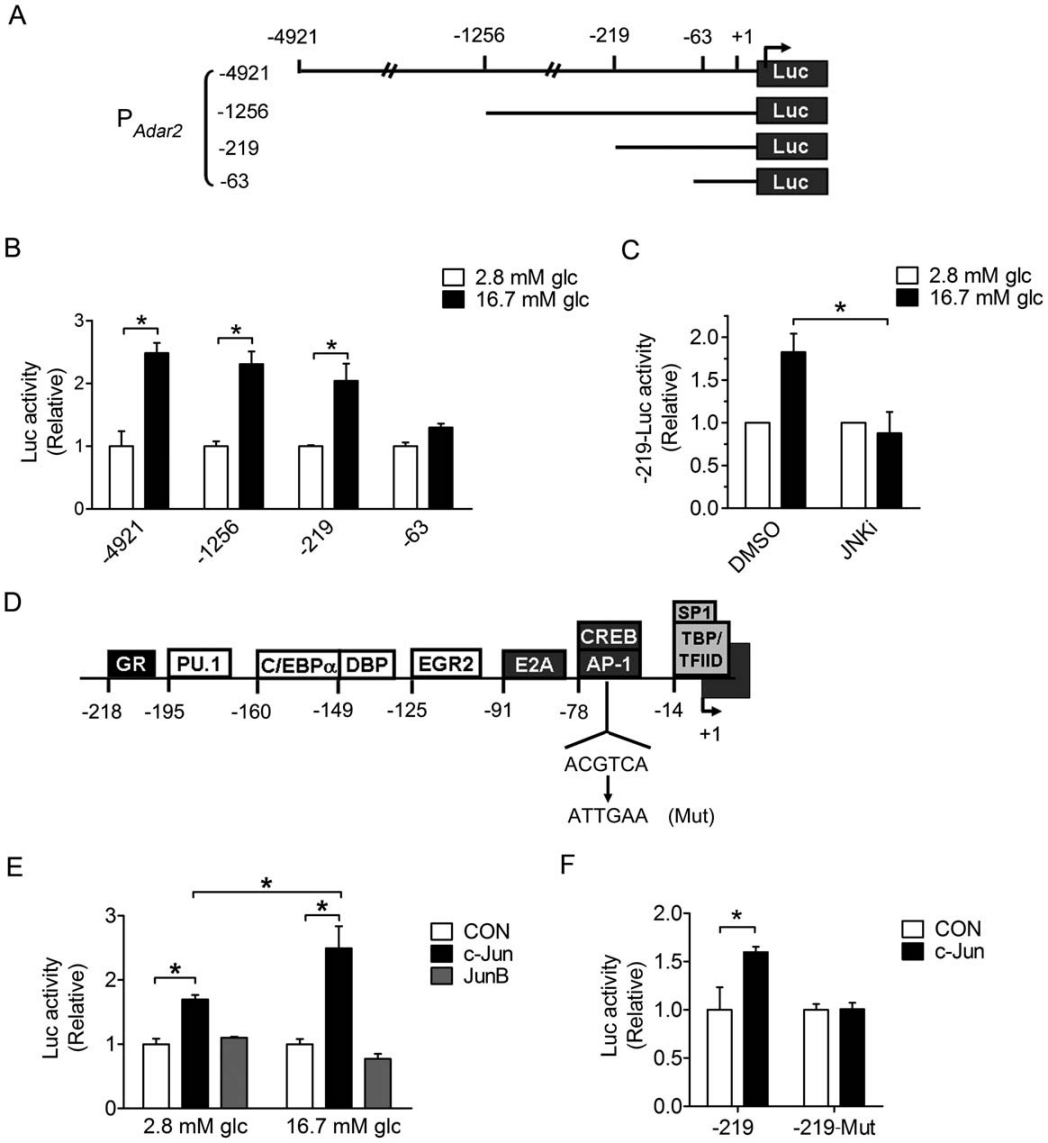
Analysis of the mouse *Adar2* promoter that confers JNK1-dependent responsiveness to glucose stimulation. (**A**) Schematic of pGL3-luciferase (Luc) reporter constructs containing the indicated regions of the mouse *Adar2* promoter. +1, the putative transcription start site. (**B**) Analysis of the transcriptional activities of the mouse *Adar2* promoter in response to glucose stimulation. INS-1 cells transfected with the indicated reporter constructs were incubated at 2.8 or 16.7 mM glucose for 16 hours. (**C**) Inhibition of JNK diminished the glucose-stimulated transcriptional activity of the *Adar2* promoter. INS-1 cells were transfected with P*_Adar2_*-219-Luc that contained the 219-nucleotide glucose-responsive region of the *Adar2* promoter. Cells were subsequently cultured at 2.8 mM or 16.7 mM glucose in the absence or presence of 20 µM JNK inhibitor SP600125 for 24 hours. For (**B**) and (**C**), promoter activities were quantified by the luciferase assay. Shown are fold increases in the luciferase activity upon high glucose stimulation after normalization to *Renilla*. Data are expressed as the mean±SEM from more than three experiments. *P<0.05 versus basal activity at 2.8 mM glucose. (**D**) Potential regulatory elements in the 219-nucleotide glucose-responsive region of the mouse *Adar2* promoter. Putative binding sites for *trans*-factors are shown. GR, glucocorticoid receptor; C/EBP, CCAAT/enhancer-binding protein; DBP, D site of albumin promoter-binding protein; EGR2, early growth response 2; CREB, cAMP-response element binding protein; AP-1, activating protein 1; SP1, specificity protein 1; TBP, TATA box-binding protein; TFIID, transcription factor II D. The core sequence for CREB/AP-1 binding is shown, and the substitution mutations are indicated for the mutant (Mut) version used for luciferase assays in (**F**). (**E**) Overexpression of c-Jun but not JunB increased the transcriptional activity of the 219-nucleotide *Adar2* promoter. INS-1 cells were co-transfected with empty control vector (CON) or the plasmid expressing c-Jun or JunB together with the pGL3-P*_Adar2_*-219 reporter construct. Luciferase activities were analyzed after incubation at 2.8 mM or 16.7 mM glucose for 18 hours. (**F**) Disruption of the putative CREB/AP-1 binding sequence abolished c-Jun activation of the 219-nucleotide *Adar2* promoter. INS-1 cells were co-transfected with empty control vector (CON) or the c-Jun plasmid together with the pGL3-P*_Adar2_*-219 or pGL3-P*_Adar2_*-219-Mut reporter construct. Cells were cultured at 11.1 mM glucose for 16 hours prior to luciferase analysis. For (**E–F**), data were normalized to *Renilla* luciferase activity and are shown as the mean±SEM from three independent experiments. The ratio between Firefly and *Renilla* luciferase activities in control cells was set as 1. *P<0.05 versus cells transfected with control vector.

Because JNK kinases exert their physiological functions through regulating transcription factors such as c-Jun, we investigated if the JNK pathway mediates glucose stimulation of ADAR2 at the transcription level in β-cells. We characterized the glucose-responsive region within the mouse *Adar2* promoter in INS-1 cells through generating a series of luciferase reporter constructs (Fig. 7A). Transient transfection and luciferase assays identified a 219-nuceotide region (P*_Adar2_*-219) that retained full capacity for glucose induction (Fig. 7B), which exhibited comparable transcriptional responsiveness to glucose stimulation to that of the 410-nuceotide rat *Insulin 1* promoter (RIP) (Figure S2). Importantly, inhibition of the JNK activity by 20 µM JNKi in INS-1 cells dramatically blunted the glucose induction of the transcriptional activity of P*_Adar2_*-219 (Fig. 7C). To further examine the potential trans-factors that may be involved in JNK-mediated glucose regulation of ADAR2, we analyzed the 219-nucleotide sequence that conferred the glucose-responsive property using TRANSFAC [40] and TESS programs (http://www.cbil.upenn.edu/cgi-bin/tess/tess). Among several putative binding sites for transcription factors, there exists a potential CREB/AP-1 (activating protein-1) binding site (ACGTCA) in the -78/−73 region of the mouse *Adar2* promoter that may account for the stimulatory effect of JNK1. Supporting this idea, co-transfection experiments showed that expression of c-Jun, but not JunB that is not phosphorylated by JNKs [41], significantly enhanced both the basal and glucose-stimulated transcriptional activity of P*_Adar2_*-219 (Fig. 7E); moreover, c-Jun was unable to activate the mutant version of P*_Adar2_*-219 (Fig. 7F), in which this AP-1 site was mutated to ATTGAA (Mut). Thus, these results implicated the JNK-c-Jun pathway in the glucose-responsive control of *Adar2* transcription in β-cells.

## Discussion

In the current study, we aimed to explore the molecular mechanism by which the RNA editing enzyme ADAR2 in pancreatic β-cells or primary islets is regulated in response to glucose stimulation or changes in the nutritional state. We demonstrated that the stress-activated protein kinase JNK1 is a crucial signaling molecule in mediating the metabolic control of the transcriptional expression of ADAR2 in β-cells. These findings provide new insight with respect to the mechanism for cell type-specific modulation of ADAR2 expression and ADAR2-dependent RNA editing under physiological conditions.

RNA editing by ADAR2 is known to play essential roles in the central nervous system [12], [42], [43]. However, limited knowledge has been obtained regarding the functions and regulation of ADAR2 in the periphery. We previously found that in professional secretory cells such as pancreatic β-cells, ADAR2 expression is regulated in response to nutritional and metabolic cues [33] and its deficiency can influence the cellular exocytotic output [34]. In the state of excess energy intake, ADAR2 may be connected to overnutrition-associated chronic inflammatory pathways. The JNK signaling cascade is an ancient stress pathway that mediates cellular adaptation responses to environmental changes, thereby promoting the survival of an organism [44]. On the other hand, chronic activation of the JNK pathway is known to play an important role in the pathogenesis of obesity-related insulin resistance and metabolic dysfunctions, including impairment of β-cell function in diabetes [29], [45], [46]. Here our results indicate that activation of the JNK pathway is metabolically coupled to the glucose-dependent upregulation of ADAR2, which occurs in a manner that requires the metabolism of glucose. The results from the islets of HFD-fed mice further suggest that JNK1-mediated regulation of ADAR2 expression may represent a unique aspect of the nutrient-sensing events in β-cells, and glucose may be one of the various metabolic signals that contribute to JNK1 activation and ADAR2 upregulation in the state of overnutrition. In this scenario, it remains to be investigated whether JNK signaling exerts its deleterious effect upon β-cell functions through affecting ADAR2-mediated RNA editing actions, particularly under the condition of obesity-related metabolic stress.

Gene-targeting studies in mice have revealed distinctive as well as redundant functions of the different JNK isoforms [46]. Deficiency of JNK1 but not JNK2 was reported to result in reduced adiposity and steatohepatitis as well as improved insulin sensitivity in mouse models of obesity [29], [45]. While studies in pancreatic islets suggested important roles for both JNK1 and JNK2 in the survival and function of β-cells [32], [47], the precise metabolic action of each JNK isoform is yet to be delineated. We observed that JNK1, instead of JNK2, mediates the glucose-responsive upregulation of ADAR2 expression, providing another example for JNK isoform-selective regulatory action. In addition, JNK3 was shown to be expressed in human pancreatic islets, but given that knockdown of JNK3 increased c-Jun levels [48], it is unclear whether JNK3 contributes to the glucose-responsive regulation of ADAR2 in pancreatic β-cells.

Of note, it has been shown that JNK1 phosphorylates the transcription factor c-Jun for its activation, whereas JNK2 lacks this activity [49], [50]. Consistently, we found that the mouse *Adar2* promoter contains several potential binding sites for trans-factors including AP-1, which is likely to link JNK1 signaling to the upregulation of *Adar2* transcription. The AP-1 family of transcription factors comprise homodimers and heterodimers of Jun, Fos or activating transcription factor (e.g. ATF2) protein [28]. It has been established that Jun-Jun and Jun-Fos dimers bind preferentially to a 12-O-tetradecanoylphorbol-13-acetate-responsive element (TGACTCA), whereas the Jun-ATF dimer can bind to the cAMP-responsive element (TGACGTCA) [51]. As ATF2 can also be directly phosphorylated by JNK [52], it remains to be deciphered whether a c-Jun-ATF2 complex is involved in mediating JNK1-dependent regulation of ADAR2 expression. Furthermore, as suggested by the presence of other putative sequence elements within the *Adar2* promoter for transcriptional regulators such as glucocorticoid receptor (GR), CCAAT/enhancer-binding protein α (C/EBPα) or D site of albumin promoter-binding protein (DBP), ADAR2 may be transcriptionally controlled in the context of a wide range of biological processes that remain to be defined.

The glucose-sensing mechanism is employed by many endocrine cells to control the secretion of hormones, which is best exemplified by glucose stimulation of insulin secretion and inhibition of glucagon secretion in pancreatic islets [53]. Moreover, glucose also regulates the activity of glucose-responsive neurons that are present in the brain stem and hypothalamus [54]. The glucose-excited or -inhibited neurons respond to changes in the local glucose concentration and play critical roles in glucose and energy homeostasis [53], [55]. Documented studies in the brain have implicated ADAR2 in the control of energy balance [56], and it remains enigmatic whether ADAR2 exerts its actions in glucose-responsive neurons. In addition, many of the ADAR-targeted substrate RNAs encode key components of neurotransmission that are known to play crucial roles in the control of glucose and energy homeostasis, e.g. serotonin receptor-2C [57], [58] and GABA [59]. Given the functional importance of JNK1 in the brain with respect to metabolic control [60], [61] as well as the widespread A to I RNA editing events as recently revealed by the human editome analysis [62], it remains an open question whether the JNK pathway can respond to nutritional changes and exerts its regulatory action upon ADAR2-dependent RNA editing in specific nutrient-sensing neurons. As the JNK signaling cascade has been implicated in various physiological and pathological processes including inflammation, metabolic disorders and neurodegenerative diseases [46], [63], [64], the functional connection between the JNK pathway and ADAR2-mediated RNA editing warrants further investigations.

## Supporting Information

Figure S1 **JNK1 deletion protects mice against hyperglycemia resulting from HFD feeding.** After a fast of 6 hours, blood glucose concentration was determined for JNK1^−/−^ mice and their WT littermates, which were fed HFD or LFD for 16 weeks (n = 5-7/group). Data are shown as the mean±SEM. *P<0.05, **P<0.01.
(TIF)

Figure S2 **The 219-nucleotide region of the mouse** ***Adar2*** **promoter has similar glucose-responsive transcription activity as that of the rat insulin promoter.** INS-1 cells were transfected for 40 hours with the P*_Adar2_*-219-Luc or the 410-nucleotide RIP-Luc reporter constructs. Luciferase activities were analyzed after cells were cultured at 2.8 mM or 16.7 mM glucose for 16 hours. Shown are fold increases in the luciferase activities upon high glucose stimulation after normalization to *Renilla* luciferase activity. Data are presented as the mean±SEM from three independent experiments. *P<0.05.
(TIF)

Table S1 **The oligonucleotide sequences of primers used.**
(DOC)
