# Supplementary figures and images for: c-Jun Amino-Terminal Kinase-1 Mediates Glucose-Responsive Upregulation of the RNA Editing Enzyme ADAR2 in Pancreatic Beta-Cells
Source: PLoS One. 2012 Nov 6;7(11):e48611. doi: 10.1371/journal.pone.0048611 (PMC3490865; doi:10.1371/journal.pone.0048611)

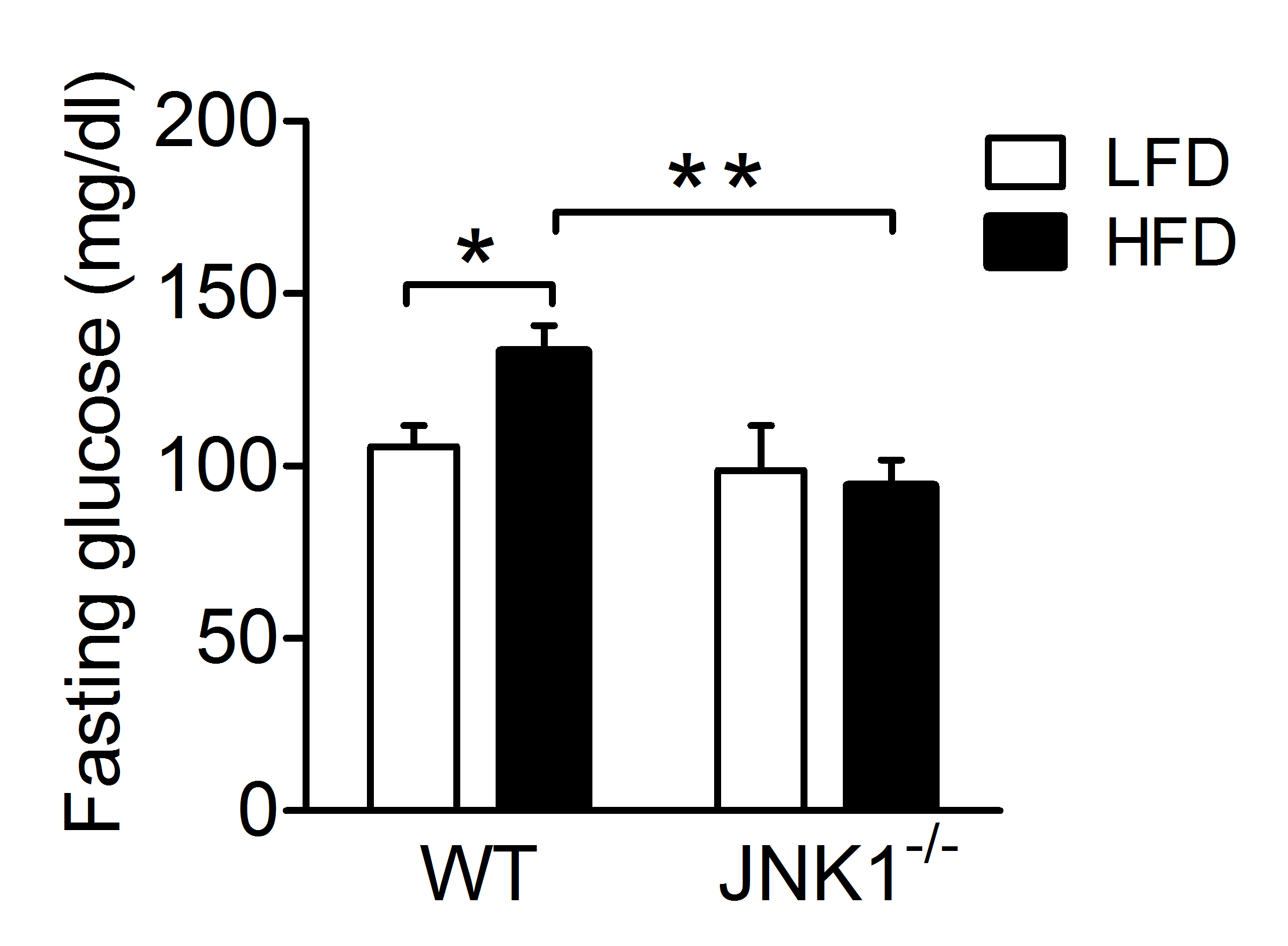

Supplement: Figure S1 — JNK1 deletion protects mice against hyperglycemia resulting from HFD feeding. After a fast of 6 hours, blood glucose concentration was determined for JNK1−/− mice and their WT littermates, which were fed HFD or LFD for 16 weeks (n = 5-7/group). Data are shown as the mean±SEM. *P<0.05, **P<0.01. (TIF) [file pone.0048611.s001.tif]

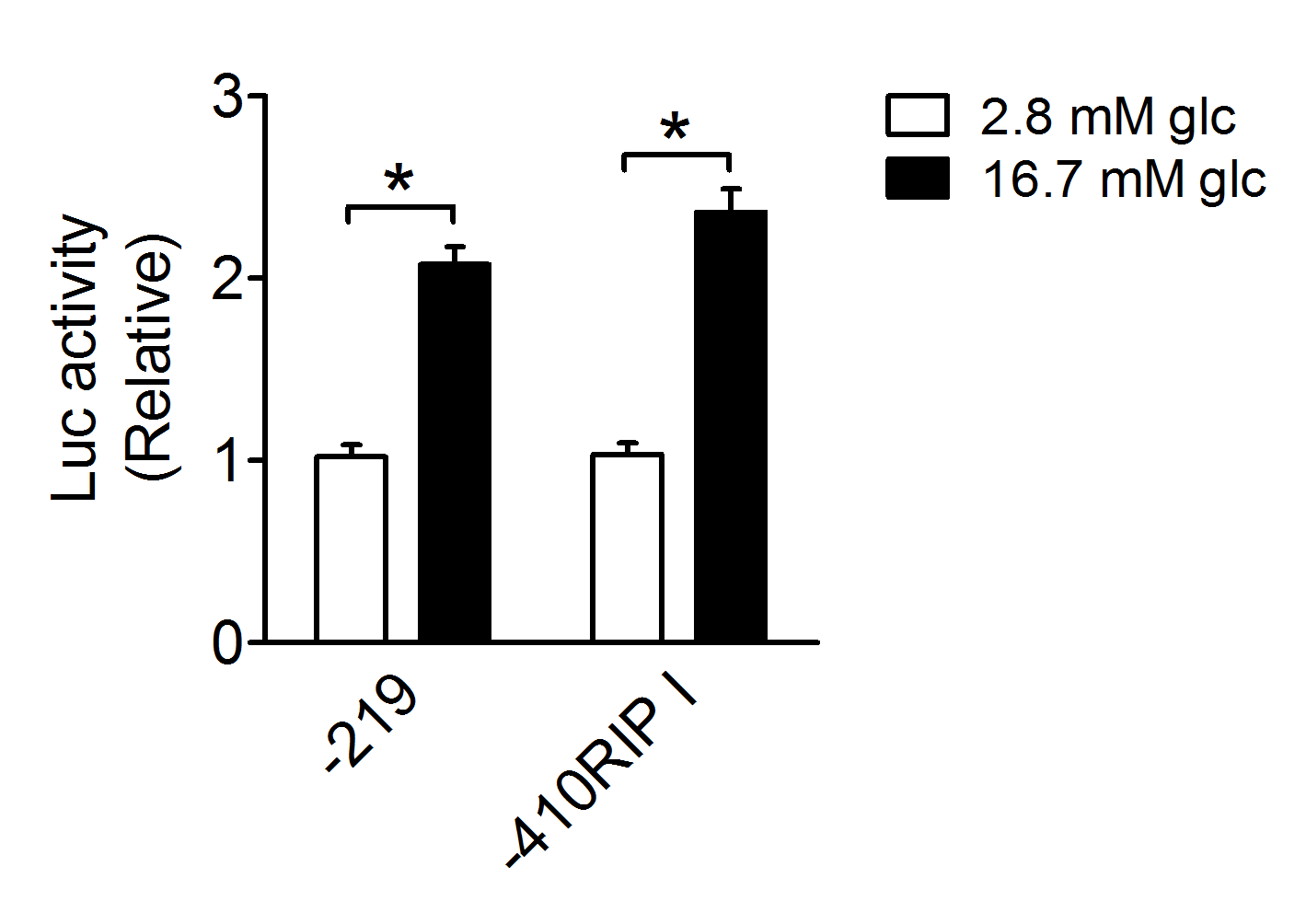

Supplement: Figure S2 — The 219-nucleotide region of the mouse Adar2 promoter has similar glucose-responsive transcription activity as that of the rat insulin promoter. INS-1 cells were transfected for 40 hours with the PAdar2-219-Luc or the 410-nucleotide RIP-Luc reporter constructs. Luciferase activities were analyzed after cells were cultured at 2.8 mM or 16.7 mM glucose for 16 hours. Shown are fold increases in the luciferase activities upon high glucose stimulation after normalization to Renilla luciferase activity. Data are presented as the mean±SEM from three independent experiments. *P<0.05. (TIF) [file pone.0048611.s002.tif]
