# Supplementary material for: c-Jun Amino-Terminal Kinase-1 Mediates Glucose-Responsive Upregulation of the RNA Editing Enzyme ADAR2 in Pancreatic Beta-Cells
Source: PLoS One. 2012 Nov 6;7(11):e48611. doi: 10.1371/journal.pone.0048611 (PMC3490865; doi:10.1371/journal.pone.0048611)
Supplement: Table S1 — The oligonucleotide sequences of primers used. (DOC) [file pone.0048611.s003.doc]

**Table S1: The oligonucleotide sequences of primers used.**

| **RT-PCR analysis for rat and mouse ADAR2 auto-editing** |  |
| --- | --- |
| Forward primer | 5'-AAAAGAGGTCTCCGCCAGTC-3' |
| Reverse primer | 5'-TTCGCTTCTTCAGGCGGTAC-3' |
|  |  |
| **Quantitative real-time RT-PCR** |  |
| Rat ADAR1 (+) | 5'-GAGCAGTTGGGTTTCGCAG-3' |
| Rat ADAR1 (-) | 5'-AAACTGTTGGTCAGAGCGTTGAAG-3' |
| Rat ADAR1-p150 (+) | 5'- CACAGACCCAGTCACTACCG -3' |
| Rat ADAR1-p150 (-) | 5'- AGCAAAGACCTTCAGCACCT -3 |
| Rat ADAR2 (+) | 5'-TCAATTTGCACTTGGACCAAAC-3' |
| Rat ADAR2 (-) | 5'-CAGGCGTGAGACAGCATCTG-3' |
| Rat JNK1 (+) | 5'-GGCAGCCGTCTCCTTTAGC -3' |
| Rat JNK1 (-) | 5'-TGTATCCGAGGCCAGAGTCG-3' |
| Rat JNK2 (+) | 5'-AAGCACCTGCATTCAGCTGG -3' |
| Rat JNK2 (-) | 5'- AGTTGGTACAGGCTGTCCGTG-3' |
| Rat GAPDH (+) | 5' -GGATTTGGCCGTATCGG-3' |
| Rat GAPDH (-) | 5’-GTTGAGGTCAATGAAGGGG-3’ |
| Mouse and rat actin (+) | 5'-GGCCAACCGTGAAAAGATGA-3' |
| Mouse and rat actin (-) | 5'-GACCAGAGGCATACAGGGACA-3' |
| Mouse and rat cyclophilin (+) | 5'-ATGGCAAATGCTGGACCAAA-3' |
| Mouse and rat cyclophilin (-) | 5'-CATGCCTTCTTTCACCTTCCC-3' |
| Mouse ADAR2 (+) | 5'-TGTAAGCACGCGCTGTACTGT-3' |
| Mouse ADAR2 (-) | 5'-GACTCGTGGTATGTGGTAGGCTTAG-3' |
| Mouse ADAR1 (+) | 5'-CCAACCCCGTAGGCGG-3' |
| Mouse ADAR1 (-) | 5'-CCAACTTTTGCTTGGTAAACAAACT-3' |
| Mouse ADAR1-p150 (+) | 5’ CACTATGTCTCAAGGGTTCAGGG 3’ |
| Mouse ADAR1-p150 (-) | 5’ CACTTGCTATGCTCATGACTAGGG 3’ |
| Mouse JNK1 (+) | 5'-AATGCTGTGTGGAATCAAGCAC-3' |
| Mouse JNK1 (-) | 5'-CCTCGCCAGTCCAAAATCAA-3' |
| Mouse JNK2 (+) | 5'-AGCGGCTTTCTCTCTCCCC-3' |
| Mouse JNK2 (-) | 5'-TCATAGGTGCACTTGCCTGG-3' |
